# Injured submucosal arteries following cold snare polypectomy are significantly fewer versus those after endoscopic mucosal resection for 10–19‐mm nonpedunculated colorectal polyps

**DOI:** 10.1002/deo2.70099

**Published:** 2025-03-18

**Authors:** Shingo Kurasawa, Ichitaro Horiuchi, Masashi Kajiyama, Hiroe Kitahara, Tsuyoshi Terashima, Akira Horiuchi

**Affiliations:** ^1^ Department of Pediatrics Shinshu University School of Medicine Nagano Japan; ^2^ Digestive Disease Center Showa Inan General Hospital Nagano Japan; ^3^ Department of Surgery Showa Inan General Hospital Nagano Japan; ^4^ Department of Pathology Showa Inan General Hospital Nagano Japan

**Keywords:** cold snare polypectomy, colorectal polyp, delayed bleeding, endoscopic mucosal resection, injured submucosal artery

## Abstract

**Objectives:**

We compared the frequency of post‐polypectomy bleeding or injured submucosal arteries between cold snare polypectomy (CSP) and endoscopic mucosal resection (EMR) for nonpedunculated colorectal polyps.

**Methods:**

This was a prospective, randomized, single‐center study. Patients who underwent CSP or EMR for 10–19‐mm nonpedunculated polyps were enrolled in CSP and EMR groups, and we compared the patient and polyp characteristics, the number of clips used, clinical outcomes, adverse events, and pathological features of the resected polyps between these groups. The primary outcome was the presence of injured arteries in the submucosal layer of the resected polyps examined histologically. The secondary outcomes were immediate bleeding and delayed bleeding.

**Results:**

Fifty‐three patients with 60 eligible polyps were enrolled. The numbers of polyps/patients were 30/26 in the CSP group and 30/27 in the EMR group. The patient and polyp characteristics were similar between the groups. The total number of hemostatic clips used for hemostasis or prophylactic clipping was significantly greater in the EMR group compared to the CSP group (78 vs. 10, *p* < 0.001). The frequency of immediate bleeding after CSP was similar to that after EMR [6.7% (2/30) vs. 13% (4/30), *p* = 0.39]. Delayed bleeding did not occur in either group. The presence of injured submucosal arteries after CSP was significantly less frequent than that after EMR: 10% (3/30) versus 67% (20/30), *p* < 0.001.

**Conclusions:**

In the resection of 10–19‐mm nonpedunculated colorectal polyps, CSP may decrease post‐polypectomy bleeding without prophylactic clipping compared to EMR as it results in fewer injured submucosal arteries. www.clinicaltrials.gov (NCT05930041).

## INTRODUCTION

The application of colorectal polypectomy techniques including the cold snare polypectomy (CSP) and endoscopic mucosal resection (EMR) techniques may have contributed to the decrease in colorectal cancer mortality.^1^ Colorectal polyps < 10 mm were reported to account for approx. 90% of all colorectal polyps,^2^ and the colorectal polypectomy guidelines in the United States and in European countries have recommended the CSP technique for the resection of <9 mm nonpedunculated colorectal polyps.^3^, ^4^ For the resection of 10–19‐mm nonpedunculated colorectal polyps, the US consensus guidelines recommend either an EMR or a cold‐snare EMR.^4^

Compared to an EMR, a CSP is a less time‐consuming and simpler surgery with a lower rate of post‐polypectomy bleeding for small colorectal polyps. A CSP using a dedicated cold polypectomy snare has been reported to result in a low rate of post‐polypectomy bleeding even in patients who are under treatment with an antithrombotic agent.^5^, ^6^ Because CSP using a dedicated cold polypectomy snare resulted in a significantly lower rate of injured submucosal arteries compared to the use of a traditional polypectomy snare.^7^ Performing a CSP can thus safely remove < 10‐mm colorectal polyps without requiring the cessation of patients' antithrombotic agents, although patients need to stop taking direct oral anticoagulants on the procedure day.^8^ We conducted the present study to compare both the frequency of postpolypectomy bleeding and the numbers of injured submucosal arteries between CSP and EMR in resections of 10–19‐mm nonpedunculated colorectal polyps.

## METHODS

### Study design

This was a prospective, randomized, open‐label, single‐center study conducted at Japan's Showa Inan General Hospital. This study was approved by the hospital's ethics committee on April 21, 2021 (No. 2021‐02) and was conducted in accordance with the Helsinki Declaration. All of the enrolled patients gave their written informed consent for their data to be published when the procedure was scheduled. The study was registered at www.clinicaltrials.gov (NCT05930041).

### Study population

Among 2567 patients who were referred to our hospital and scheduled for screening, surveillance, or diagnostic colonoscopy between January and July 2023, those who were 18–80 years of age and had at least one 10–19‐mm polyp (Paris classification Is or IIa) revealed by endoscopic examination were enrolled. The exclusion criteria were: (i) American Society of Anesthesiologists status class 3 or above, (ii) poor bowel preparation (Boston Bowel Preparation Scale < 6 points), (iii) endoscopic features indicating submucous infiltration or malignancy, (iv) known blood coagulation disorders, or bleeding tendency, (v) a history of colorectal resection, (vi) a history of emergent colonoscopy, (vii) inflammatory bowel disease, familial polyposis, or colorectal cancer, (viii) pregnancy or lactation, and (ix) severe cardiopulmonary dysfunction, cirrhosis, chronic kidney disease, other malignant tumors, or severe infectious diseases.

### Procedures

All procedures were conducted under propofol mono‐sedation (AstraZeneca; Nichi‐Iko Pharmaceutical) administered by a nurse supervised by an endoscopist.^9^, ^10^ The standard bowel preparation was a polyethylene glycol solution (EA Pharmaceutical).^11^ All colonoscopies were performed by one of the hospital's four endoscopists who each performed >500 endoscopies/year. The lateral decubitus position was used in all colonoscopies.

The videoscope (PCF‐H290ZI) was used for the colonoscopies. All colorectal polyps up to 19 mm (with the exception of tiny hyperplastic polyps in the rectum and distal sigmoid colon) were removed using either a CSP or an EMR. The snare for CSP was a dedicated cold snare (Captivator COLD snare; Boston Scientific) with a snare wire diameter of 0.30 mm. The snare used for the EMRs was a dual‐loop wire snare with an opening diameter of 33 or 15 mm (SN‐3316LX; Medico's Hirata). An ERBE ICC 200 electrosurgical unit (Amco) was used in the Endocut mode with the effect 3 current set at an output limit of 120 W and the forced coagulation current set at an output limit of 35 W for both the CSP and EMR techniques. A submucosal injection of saline solution was not administered in the CSP cases. For the EMR cases, a submucosal injection was administered with a needle, using a solution of epinephrine diluted 1:10,000 in saline; no contrast agent was included in the injection fluid, and 2–5 mL of solution was injected for each polyp.

After the resection using either a CSP or EMR, the absence of visible residual polyp tissue was confirmed with the use of narrow‐band imaging, and the completeness of the polyp resection was confirmed endoscopically. If residual polyp tissue was seen at the polypectomy site, it was resected. The transected small polyps were sucked into a trap. Larger polyps were retrieved using retrieval forceps without the use of the endoscopic suction channel, in order to avoid fragmenting the sample.

### Data collection

The patient monitoring data and adverse events were prospectively recorded by a registered nurse. The indications for antithrombotic agents used, the snare used, the size, location, shape, and pathology of all polyps, and the number of hemostatic clips (long clip HX‐610‐090L; Olympus) used were also prospectively recorded. Hemostatic clipping for both CSP and EMR was done for immediate bleeding or at the endoscopists' discretion. Prophylactic clipping for CSP was not routinely performed, but prophylactic clipping for EMR was routinely performed. The procedure duration was defined as the time from the initial insertion of the endoscope to its withdrawal.

All of the patients were contacted by phone 2 weeks after the procedure to be informed of the pathological results. Adverse events and all gastrointestinal symptoms occurring within the 2 weeks after either procedure were recorded. For this prospective study, the patient characteristics, polyp characteristics, indications for antithrombotic therapy, antithrombotic agents used, snare used, number of clips used, and adverse events were all documented in the hospital's online database.

### Outcome measures

The primary outcome measure was the presence of injured submucosal arteries detected in the submucosal layer.

The secondary outcomes included the frequency of immediate bleeding and delayed bleeding requiring endoscopic treatment within 2 weeks after the polypectomy. Immediate bleeding was defined as spurting or oozing continuing for >30 s.^5^, ^6^

### Randomization and concealment

Eligible polyps were randomly assigned (1:1) to the CSP group and the EMR group. A computer‐generated randomization sequence was prepared by an investigator who had no clinical involvement in the treatment procedure and was concealed by placing the assignments in opaque, sequentially numbered envelopes. When an eligible polyp was identified during a colonoscopy, an assistant opened the envelope to reveal the assigned polypectomy technique. If more than one eligible polyp was observed in a patient, randomization was conducted for each polyp.

### Pathological examination

The use of CSP or EMR remained blinded to the pathologist (Tsuyoshi Terashima) until after all of the analyses were completed. After their removal, excised polyp specimens were mounted with pins on Styrofoam plates and fixed in 10% formalin. They were examined grossly and after being sectioned they were examined using hematoxylin and eosin staining. The resection was considered complete histologically if the vertical and lateral margins were free from neoplasia tissue. The resected specimens were specifically examined for the presence of muscularis mucosae, submucosal arteries, and injured arteries in the submucosal layer. A polyp was considered positive if a muscularis mucosae, submucosal artery, or injured submucosal artery was detected in the excised polyp, respectively.

### Statistical analyses

The calculation of the necessary sample size was based on the study's primary outcome measure. Our prior investigations revealed the presence of injured submucosal arteries in the submucosal layer following CSP or hot snare polypectomy.^5^, ^6^ Based on that experience, we hypothesized that the rate of the presence of injured submucosal arteries detected in the submucosal layer by cold snaring using the exclusive CSP snare would be <10% while that for the EMR cases would be 50%; thus, ≥20 polyps per group was required to demonstrate the presence of injured submucosal arteries in the submucosal layer in the CSP group compared with the EMR group with an alpha value of 0.05 and 80% power.

Data are presented as the median (range). The *χ* ^2^‐test was used for proportions with Yates' correction for continuity where appropriate. For parametric data, Student's *t*‐test was used when two means were compared. The factors associated with baseline characteristics and clinical outcomes of patients treated with CSP or EMR were evaluated using multivariate analysis. A *p*‐value <0.05 was regarded as significant. The statistical analyses were performed with GraphPad Prism ver. 10.1.1 software (GraphPad Software).

## RESULTS

### Patients

Fifty‐three patients (CSP, *n* = 26; EMR, *n* = 27) were enrolled. Table 1 summarizes the patients' baseline characteristics and clinical features. The patients' baseline characteristics including the use of antithrombotic agents were similar between the CSP and EMR groups (Table 1).

**TABLE 1:** Comparison of the baseline characteristics of patients treated with cold snare polypectomy (CSP) or endoscopic mucosal resection (EMR).

	CSP	EMR	*p*
Patients, *n*	26	27	
Males/females	18/8	21/6	0.54
Median age, years	77	72	0.67
Indications:			>0.99
Screening/Surveillance	13	13	
Hemo‐positive stool	10	11	
Other	3	2	
Antithrombotic agents:	2	3	0.40
Warfarin	0	1	
Clopidogrel	2	0	
Cilostazol	0	1	
Clopidogrel+cilostazol	0	1	

Abbreviations: CSP, cold snare polypectomy; EMR, endoscopic mucosal resection.

### Clinical outcomes and adverse events

Table 2 shows outcomes and adverse events in the CSP and EMR groups. All polyps were completely resected in both groups, although partial resections were performed in the CSP group. Completeness of polyp resection was confirmed endoscopically using narrow‐band imaging after two or three piecemeal resections in the CSP group. The number of patients using hemostatic clips was 7/26 (27%) in the CSP group. One clip was used for prophylactic clipping in five patients. In two patients, two or three hemostatic clips were used to stop immediate bleeding, giving a total of 10 hemostatic clips used in the CSP group. On the other hand, the number of patients using hemostatic clips in the EMR group was 27/27 (100%). Regardless of the presence of immediate bleeding, two or three hemostatic clips were used in each of the 27 patients, resulting in a total of 78 clips used in the EMR group. The total number of hemostatic clips used for hemostasis of immediate bleeding or prophylactic clipping was significantly greater in the EMR group compared to the CSP group (78 vs. 10, *p* < 0.001). The frequency of immediate bleeding after CSP was similar to that after EMR (6.7% [2/30] vs. 13% [4/30], *p* = 0.39). Each instance of immediate bleeding was successfully stopped by a single session of endoscopic intervention. No delayed bleeding occurred in either group. Although multivariate analyses were incorporated into the analyses presented in Tables 1 and 2, the sample size was too small to yield statistically significant results. There were no significant differences in these characteristics of the polyps resected between the CSP and EMR groups (Table 3).

**TABLE 2:** Comparison of outcomes and adverse events in patients treated with cold snare polypectomy or endoscopic mucosal resection.

	CSP	EMR	*p*
Total no. of colorectal polyps resected	30	30	
Median no. of polyps per patient	1.2	1.1	
Median total procedure time per patient (min)	15	18	0.66
Complete resection rate, *n* (%)	30 (100)	30 (100)	
Patients using hemostatic clips, *n* (%)	7 (27)	27 (100)	<0.001
No. of total hemostatic clips with total patients	10	78	<0.001
Postpolypectomy bleeding:			
Immediate bleeding, *n* (%)	2 (6.7)	4 (13)	0.39
Delayed bleeding, *n*	0	0	
Perforation, *n*	0	0	

Abbreviations: CSP, cold snare polypectomy; EMR, endoscopic mucosal resection.

**TABLE 3:** Comparison of characteristics of polyps resected with cold snare polypectomy or endoscopic mucosal resection.

	CSP	EMR	*p*
Total no. of colorectal polyps resected	30	30	
Size, mm:			0.58
10–14	22	19	
15–19	8	11	
Location:			0.13
Left colon	20	26	
Right colon	10	4	
Shape:			0.43
Flat	15	11	
Sessile	15	19	

Abbreviations: CSP, cold snare polypectomy; EMR, endoscopic mucosal resection.

### Pathological features of the resected specimens

**FIGURE 1:**
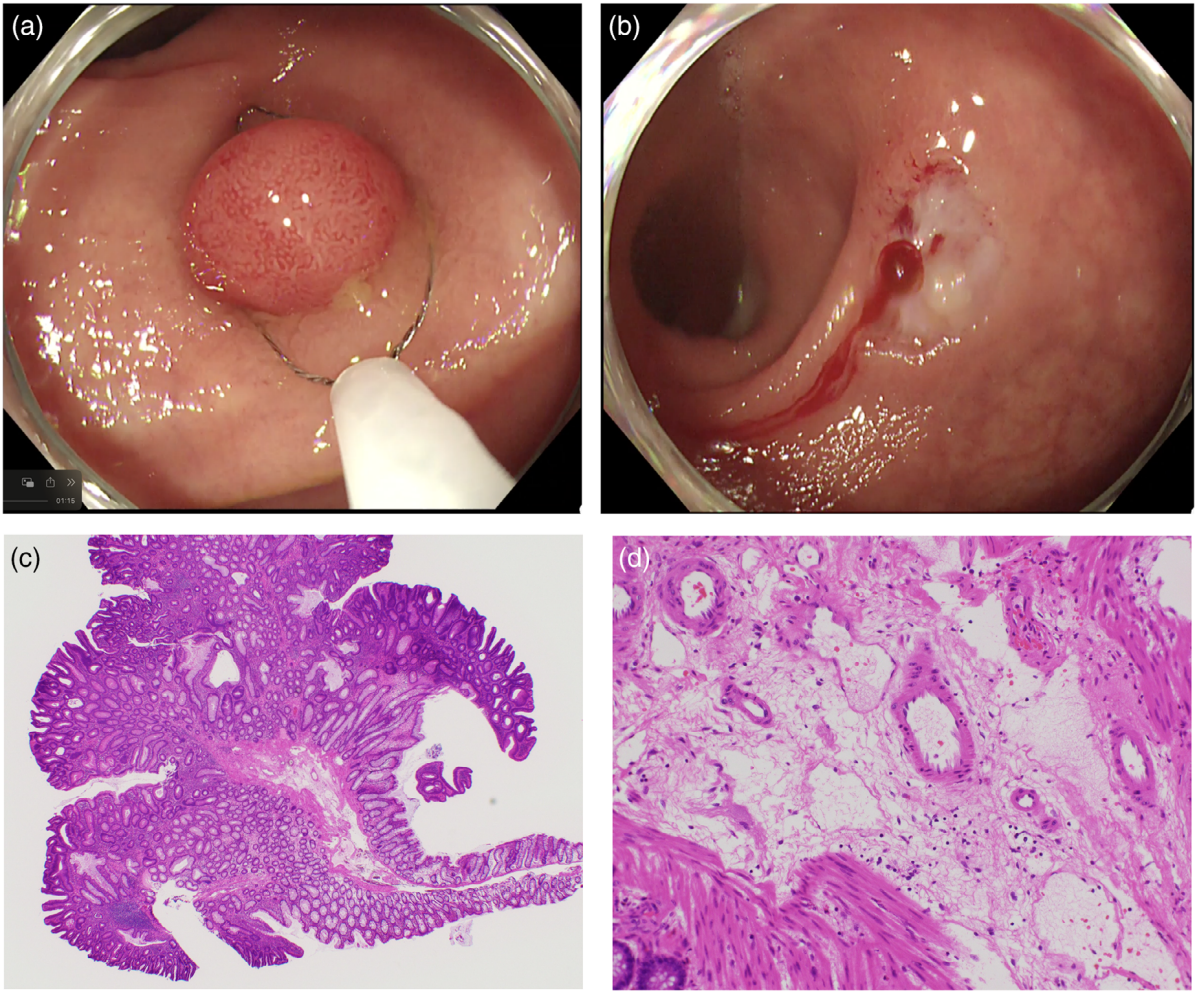
The endoscopic findings (a, b) and the pathologic specimen of a resected polyp from a patient who underwent a cold snare polypectomy. There is no injured artery in the submucosal layer (c, low resolution; d, high resolution).

**FIGURE 2:**
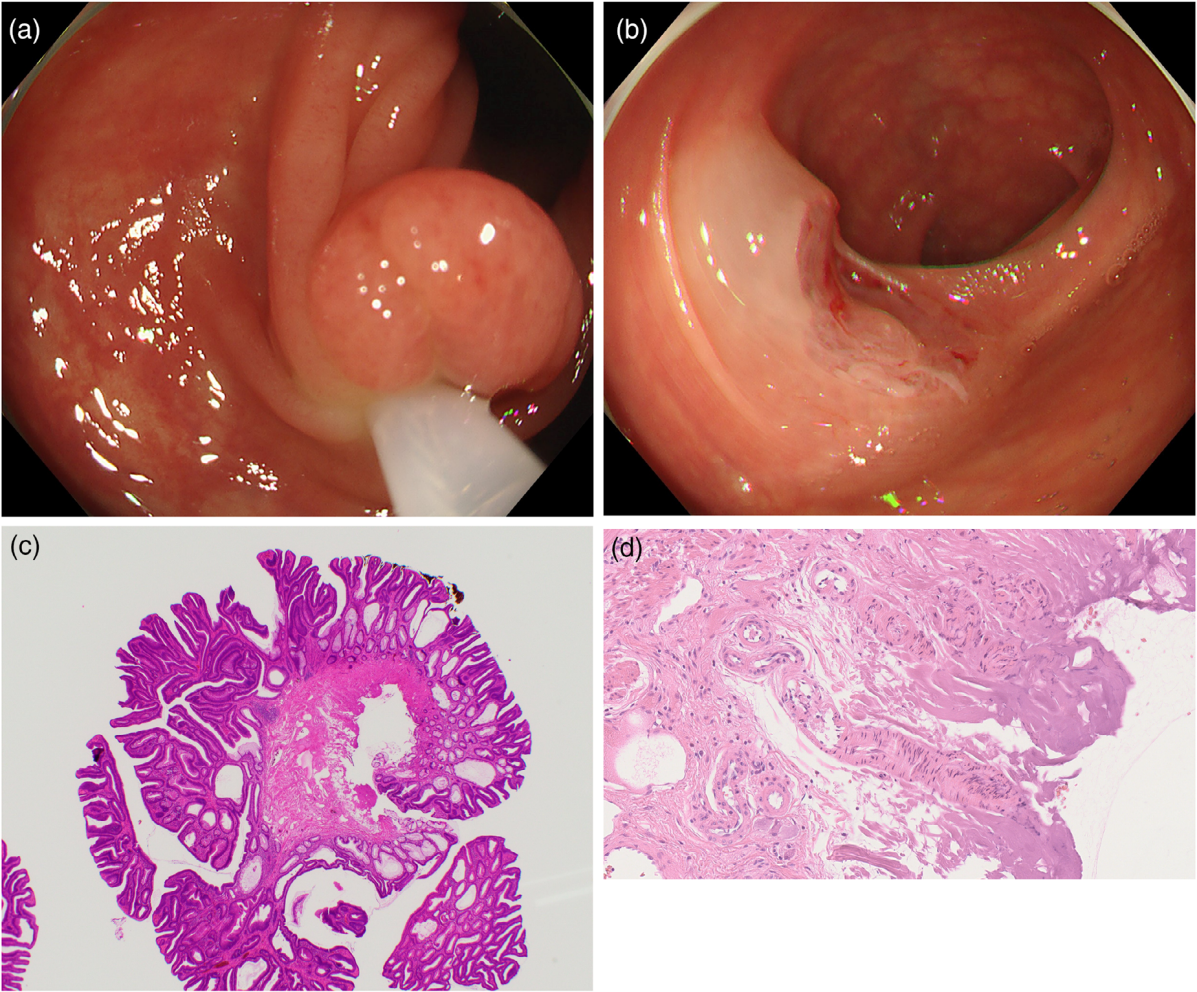
The endoscopic findings (a, b) and pathologic specimen of a resected polyp from a patient who underwent an endoscopic mucosal resection. An injured artery is present in the submucosal layer (c, low resolution; d, high resolution).

Figure 1 is of endoscopic findings (A, B) and a pathologic specimen (C, D) of a resected polyp from a representative CSP‐group patient without injured submucosal arteries in the submucosal layer, and Figure 2 is of endoscopic findings (A, B) and a pathologic specimen (C, D) of a resected polyp from a representative EMR‐group patient with injured submucosal arteries in the submucosal layer.

The pathological features of the specimens resected by CSP or EMR are summarized in Table 4. The frequency of arteries detected in the submucosa after CSP was significantly lower than that after EMR: 43% (13/30) versus 90% (27/30), *p* < 0.001. The presence of injured submucosal arteries after CSP was also significantly less than that after EMR: 10% (3/30) versus 67% (20/30), *p* < 0.001.

**TABLE 4:** Comparison of pathological features in resected specimens from patients treated with cold snare polypectomy or endoscopic mucosal resection.

	CSP	EMR	*p*
Total no. of polyps resected	30	30	
Median size of resected specimens (range), mm	14 (11–19)	15 (11–19)	0.56
Pathology:			0.078
Adenoma	20	26	
Sessile serrated lesion	6	4	
Hyperplastic polyp	4	0	
Presence of muscularis mucosae in resected polyps, *n* (%)	30/30 (100)	30/30 (100)	
Presence of submucosal arteries in resected polyps, *n* (%)	13/30 (43)	27/30 (90)	<0.001
Presence of injured submucosal arteries in resected polyps, *n* (%)	3/30 (10)	20/30 (67)	<0.001

Abbreviations: CSP, cold snare polypectomy; EMR, endoscopic mucosal resection.

## DISCUSSION

One of our research group's earlier studies demonstrated that (i) delayed bleeding requiring hemostasis occurred significantly less frequently after CSP compared to hot snare polypectomy for polyps up to 10 mm in size, despite the continuation of anticoagulants (0% vs. 14%, *p* = 0.027) and (ii) injured submucosal arteries were significantly less frequent after CSP compared to hot snare polypectomy (22% vs. 39%, *p* = 0.023).^5^ Another study described significantly less injury to submucosal arteries when a dedicated cold snare was used compared to a traditional cold snare (4.1% vs. 16%, *p* = 0.009) for polyps 6–10 mm in diameter, suggesting that the polypectomy technique, the snare used, and the polyp size may affect post‐polypectomy bleeding.^6^ The results of these previous studies roughly corresponded to those of our present investigation of 10–19‐mm nonpedunculated polyps (10% vs. 67%, *p* < 0.001). However, rather than concluding the reduction of bleeding risk solely based on pathological evidence, it is necessary to clarify the incidence of delayed clinical bleeding through follow‐up in large‐scale studies.

In routine clinical practice, delayed bleeding is more important than immediate bleeding because delayed bleeding cannot be predicted during a colonoscopy. However, the degree of immediate bleeding may predict post‐polypectomy bleeding, including delayed bleeding. In our hospital's endoscopy unit, immediate bleeding requiring hemostatic clipping is defined as spurting or oozing that continues for >30 s, and prophylactic clipping for EMR is routinely performed, although the prophylactic use of hemostatic clips has not been proven to be effective for the prevention of delayed bleeding after a conventional polypectomy, even in patients not receiving antithrombotic drugs.^12^ In the present series, the numbers of total hemostatic clips used for the hemostasis of immediate bleeding or prophylactic clipping were significantly different between the EMR and CSP groups (78 vs.10, *p* < 0.001) while the frequency of post‐polypectomy bleeding was similar between EMR and CSP (Table 2). Based on our pathology finding that the presence of injured submucosal arteries after EMR was significantly more frequent compared to that after CSP in the resected specimens (67% vs. 10%, *p* < 0.001), prophylactic clipping may prevent post‐polypectomy bleeding after EMR. The routine use of prophylactic clipping in the EMR group and its absence in the CSP group might influence outcome comparisons, possibly favoring CSP. Although additional details regarding the definition and criteria for clipping between the two groups were expected, this study could not provide more than the fact that prophylactic clipping for CSP was not routinely performed, whereas prophylactic clipping for EMR was routinely performed at the discretion of the endoscopist.

Wide‐field piecemeal CSP has been applied for non‐pedunculated large colon polyps, with or without a submucosal injection.^13^, ^14^, ^15^ CSP was reported to be safe and effective for the removal of colorectal polyps up to 15 mm in size, with a low incomplete resection rate.^16^ CSP without submucosal injection was reported to be a safe and effective treatment for sessile serrated lesions ≥ 10 mm.^17^ Cold EMR appeared to be better than CSP for complete resection of non‐pedunculated colonic polyps of 10 to 19 mm, although two techniques were not compared.^18^ In addition, underwater EMR may be the best method to contain SM tissue without injection for non‐pedunculated small polyps (endoscopically diagnosed as 6–9 mm), based on the data that the thickness of SM tissue by CSP, hot snare polypectomy, and underwater EMR were 52, 623, and 1119 µm, respectively (*p* < 0.001).^19^ These results may be compatible with the phenomenon that the presence of submucosal arteries was significantly less after CSP than after EMR (43% [13/30] vs. 90% [27/30], *p* < 0.001; Table 4).

Based on our previous studies,^5^, ^6^, ^7^ the authors believe that injury of blood vessels in the submucosal layer caused by electrocautery during EMR increases the rate of damage to the submucosal artery, making it more likely to cause delayed bleeding. On the other hand, CSP, which does not use electrocautery and does not cause thermal injury, does not damage the submucosal artery and is therefore less likely to cause delayed bleeding without prophylactic clipping. In addition, CSP without submucosal lifting may also reduce damage to the submucosal vessels. This could be resolved by having a group of CSPs with lifting, resulting in ending a debate that often occurs between the benefits of CSP with and without lifting. Therefore, it would be a subject of future research to investigate the difference in vascular injury rates between CSP and cold EMR.

This study has some limitations to address. It was conducted at a single hospital in Japan and had a relatively small sample size. Very few of the patients took direct oral anticoagulants. Delayed bleeding was not observed in any patients, and this result may be due to the small number of cases enrolled. Randomized multicenter trials of larger numbers of patients with polyps of various sizes are necessary.

In conclusion, for the resection of 10–19‐mm nonpedunculated colorectal polyps, the use of the CSP technique may decrease post‐polypectomy bleeding without prophylactic clipping compared to EMR by decreasing the presence of injured submucosal arteries. This study is limited by being a single‐center investigation with a small number of cases, which would enhance the accuracy of interpreting bleeding risk assessment.

## CONFLICT OF INTEREST STATEMENT

None

## ETHICS STATEMENT

This study was approved by the hospital's ethics committee on April 21, 2021 (No. 2021‐02).

## PATIENT CONSENT STATEMENT

All of the enrolled patients gave their written informed consent for their data to be published when the procedure was scheduled.

## CLINICAL TRIAL REGISTRATION

The study was registered at www.clinicaltrials.gov (NCT05930041).

CONSORT 2010 checklist of information to include when reporting a randomized trial is attached.

## Supporting information



CONSORT 2010 checklist of information to include when reporting a randomised trial*

## Data Availability

The data underlying this article will be shared on reasonable request to the corresponding author.
